# tsRFun: a comprehensive platform for decoding human tsRNA expression, functions and prognostic value by high-throughput small RNA-Seq and CLIP-Seq data

**DOI:** 10.1093/nar/gkab1023

**Published:** 2021-11-10

**Authors:** Jun-Hao Wang, Wen-Xin Chen, Shi-Qiang Mei, Yue-Dong Yang, Jian-Hua Yang, Liang-Hu Qu, Ling-Ling Zheng

**Affiliations:** MOE Key Laboratory of Gene Function and Regulation, State Key Laboratory for Biocontrol, Sun Yat-sen University, Guangzhou 510275, P.R. China; School of Medicine, Sun Yat-sen University, Shenzhen, Guangdong, P.R. China; MOE Key Laboratory of Gene Function and Regulation, State Key Laboratory for Biocontrol, Sun Yat-sen University, Guangzhou 510275, P.R. China; MOE Key Laboratory of Gene Function and Regulation, State Key Laboratory for Biocontrol, Sun Yat-sen University, Guangzhou 510275, P.R. China; National Supercomputer Center in Guangzhou, Sun Yat-sen University, Guangzhou, P.R. China; MOE Key Laboratory of Gene Function and Regulation, State Key Laboratory for Biocontrol, Sun Yat-sen University, Guangzhou 510275, P.R. China; MOE Key Laboratory of Gene Function and Regulation, State Key Laboratory for Biocontrol, Sun Yat-sen University, Guangzhou 510275, P.R. China; MOE Key Laboratory of Gene Function and Regulation, State Key Laboratory for Biocontrol, Sun Yat-sen University, Guangzhou 510275, P.R. China

## Abstract

tRNA-derived small RNA (tsRNA), a novel type of regulatory small noncoding RNA, plays an important role in physiological and pathological processes. However, the understanding of the functional mechanism of tsRNAs in cells and their role in the occurrence and development of diseases is limited. Here, we integrated multiomics data such as transcriptome, epitranscriptome, and targetome data, and developed novel computer tools to establish tsRFun, a comprehensive platform to facilitate tsRNA research (http://rna.sysu.edu.cn/tsRFun/ or http://biomed.nscc-gz.cn/DB/tsRFun/). tsRFun evaluated tsRNA expression profiles and the prognostic value of tsRNAs across 32 types of cancers, identified tsRNA target molecules utilizing high-throughput CLASH/CLEAR or CLIP sequencing data, and constructed the interaction networks among tsRNAs, microRNAs, and mRNAs. In addition to its data presentation capabilities, tsRFun offers multiple real-time online tools for tsRNA identification, target prediction, and functional enrichment analysis. In summary, tsRFun provides a valuable data resource and multiple analysis tools for tsRNA investigation.

## INTRODUCTION

tRNA-derived small RNAs (tsRNAs) are a novel class of functional RNA molecules that are derived from mature tRNAs or precursor tRNAs and are aberrantly expressed under various conditions (ultraviolet radiation, heat shock, hypoxia, oxidative damage or viral infection) (1–4). With the rapid advance in high-throughput sequencing technologies, many studies have reported that tsRNAs participate in essential mechanisms of cell biology, including gene regulation, transposon repression, and disease onset and progression (5–8).

The classes of tsRNA can be defined by the cleavage site position in the mature or precursor tRNA transcript (9), and include tRNA-derived stress-induced RNAs (tiRNAs) (tiRNA-5 and tiRNA-3, cleaved at the anticodon loop), and tRNA-derived fragments (tRFs) (tRF-5, cleaved at the D-loop; tRF-3, cleaved at the T-loop; tRF-i, cleaved in the internal region of the mature tRNA; and tRF-1, cleaved at the 3′ end of the tRNA precursor. Supplementary Figure S1) (10–12). Early studies reported that tiRNAs are derived from mature tRNAs through cleavage by angiogenin (ANG) and that tRFs originate from cleavage of either the mature tRNA or the tRNA precursor by Dicer or ANG (13–15).

Many studies have found that tRFs and tiRNAs can serve as novel biomarkers for disease diagnosis and prognosis. Wu *et al.* demonstrated the diagnostic value of tRF in colorectal cancer (16), and Zhu *et al.* showed the existence of abundant tsRNAs in exosomes and highlighted the diagnostic value of tsRNAs as promising biomarkers for cancer (17). TDR-000620, a tsRNA molecule, can serve as an independent adverse prognostic factor of recurrence-free survival in triple-negative breast cancer patients (18). However, studies of tsRNAs as novel cancer markers are still preliminary due to the lack of comprehensive data resources on analyses of the diagnostic value of tsRNAs.

Recent studies have also revealed that tsRNAs play important biological functions within cells by binding to diverse proteins. Goodarzi *et al.* revealed that tRFs can compete for the mRNA binding sites in YBX1 to suppress breast cancer cell growth and invasion (3). Kumar *et al.* revealed that tRFs are evolutionarily conserved and associate with AGO proteins to recognize specific RNA targets (19). Researchers identified a large number of Argonaute-tRF complexes in CLIP-seq data (19,20) and revealed that tRF-5 and tRF-3 molecules can bind to Argonaute proteins via seed-based canonical target recognition. Analysis of CLASH (crossing, ligation and sequencing of chimeras) data (21) suggested that tRF-5 and tRF-3 molecules can interact with thousands of mRNAs in human cells. However, existing CLIP-seq analysis tools are mainly aimed at investigating microRNAs (miRNAs) and their targets, ignoring the relationship between intracellular tsRNAs and their target molecules.

MicroRNAs and tsRNAs are critical small RNAs in cells and can regulate gene expression by targeting mRNAs, but it remains unclear whether there are competitive or synergistic relationships between them. Studies have suggested that target genes can be repressed by both tsRNAs and miRNAs. For example, 10% of genes targeted by AGO-bound miRNAs were found to be also targeted by tsRNAs (22). The interaction network among tsRNAs, miRNAs and mRNAs can be supported by the ceRNA hypothesis. Therefore, it is important to develop an analysis tool to explore the regulatory networks composed of tsRNAs, miRNAs, and mRNAs.

In this study, we developed tsRFun, a multifunctional platform, comprising database and web-server tools, which has the following purposes. The database contains (i) an exploration of the expression patterns and prognostic value of tsRNAs in multiple cancer types; (ii) the identified relationships between tsRNAs and their target genes; (iii) the constructed tsRNA, mRNA and miRNA interaction networks and (iv) the predicted functions of tsRNAs revealed by enrichment analysis of tsRNA targets. The tsRFun web server is equipped with three online tools: (i) tsRFinder, for identifying tsRNAs and quantifying their expression from small RNA-seq data; (ii) tsRTarget, for identifying tsRNA targets and investigating the cooperative or competitive relationships between tsRNAs and miRNAs from AGO CLIP sequencing and CLASH/CLEAR data and (iii) tsRFunction, for predicting the biological functional effects of tsRNAs in 15 types of gene sets.

tsRFun provides a systematic data analysis platform to comprehensively identify and analyse the molecular features, expression patterns, target molecules, and interaction networks of tsRNAs. As a whole, tsRFun integrates abundant data resources and offers multiple developed analytical tools, providing reliable support for comprehensively revealing the roles of tsRNAs in physiological and pathological processes.

## MATERIALS AND METHODS

### Data collection and pre-processing

tsRFun integrated multiple high-throughput sequencing datasets, namely, 10 572 small RNA-seq datasets, 381 AGO CLIP datasets, and 24 CLASH/CLEAR data. The RNA-seq datasets were retrieved from The Cancer Genome Atlas database (TCGA, 32 cancer types), the CLIP and CLASH/CLEAR data were downloaded from the SRA database. We processed the data by applying quality control filters and removing sequencing adapters by Cutadapt (Version 2.10) (23) and fastp (Version 0.20.1) (24).

### Gene annotation

Human genome sequences were obtained from the UCSC bioinformatics website (25) (Version hg38), miRNA genes were downloaded from the miRBase database (26) (Release 22), and tRNA sequences were downloaded from the GtRNAdb database (27) (Release 18.1). Mature tRNA sequences were obtained by removing intron sequences and adding the ‘CCA’ tail at the end of the original sequences of the tRNAs. Fifty-nucleotide sequences downstream of tRNAs were extracted from the reference genome based on their genomic coordinates. The tRNA modification sites were retrieved from RMBase (28) (Release 2.0), a database that contains RNA modifications identified from high-throughput sequencing datasets.

### Identification of tsRNAs from small RNA-seq data

**Figure 1.**
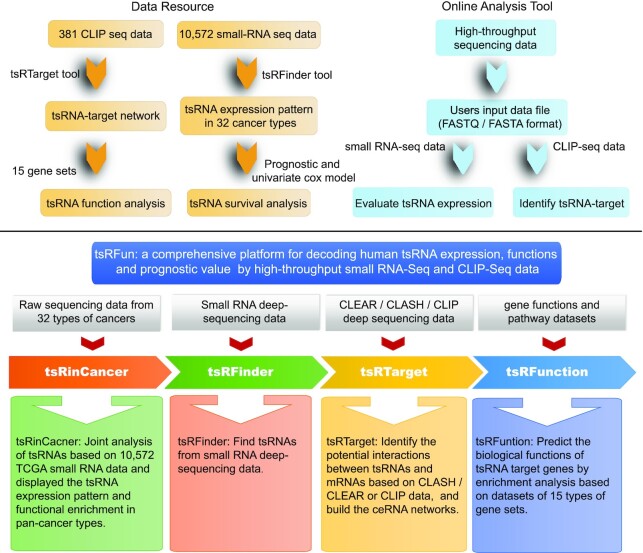
The workflow of tsRFun. The flowchart is divided into upper and lower parts. The left of the upper part shows the process of database establishment, and the right part shows the process of the online analysis tool. The lower part shows the four functional modules of the tsRFun platform and the specific description of each module.

The workflow of the tsRFun platform is shown in Figure 1. After preprocessing the sequencing data, small RNAs were mapped to the human genome to remove exogenous RNAs. Sequencing reads that were successfully mapped to known RNA transcripts (mRNAs, snoRNAs, snRNAs, rRNAs, miRNAs, or repeat sequences) were discarded. The remaining reads were then mapped to precursor and mature tRNA transcripts. We computed the *P* value of each position in tRNA transcripts according to the binomial distribution and selected the sites with significant enrichment of small RNAs with a *P* value < 0.01 (29).

The distribution of sequencing reads across the transcripts is assumed to be random or unbiased; therefore, the probability (*p*) of fragments being located at a specific 1-nt position in a certain transcript equals 1/(*L* − l + 1), where *L* and l are the lengths of the target transcript and sequencing read, respectively (30). Therefore, the probability of k or more fragments being located at a position of interest follows a binomial distribution:

(1) $$\begin{equation*}P\;\left( {X \ge k} \right) = \;\mathop \sum \limits_{x\; = \;k}^n \left( {\begin{array}{@{}*{1}{c}@{}} n\\ x \end{array}} \right){p^x}{\left( {1 - p} \right)^{n - x}}\end{equation*}$$

where *k* is the observed count of tags assigned to a given position in the mature or precursor sequence of the target tRNA, and n is the total number of tags mapped to the target transcript. Here, *P* represents the probability that the sRNA tends to be generated at a particular position. A low *P* value suggests high confidence of bona fide tsRNAs at a particular position in the tRNA transcript. Finally, the tsRNA candidates were classified into different types (tiRNA-5, tiRNA-3, tRF-5, tRF-3, tRF-i and tRF-1) according to the position of the cleavage site. Since tRNAs are conserved across the genome and fragments with identical sequences are derived from multiple tRNAs, we uniformly named the identified tsRNAs considering sequence and tRNA type (tsRNA + amino acid type + tsRNA type + unique code). For example, tsRNA-Ala-3-0055, with the sequence TCCCCGGCATCTCCACCA, was derived from position 58–75 in tRNA-Ala-CGC-1–1 and tRNA-Ala-CGC-2–1.

As we know, RNA modifications in tsRNAs can interfere with adapter ligation and reverse transcription processes during small RNA library construction and thus prevent the detection of tsRNAs bearing these modifications. Researchers have made efforts to develop special experimental methods to overcome this limitation (31). Recently Shi *et al.* have developed a novel method, PANDORA-Seq (32), to efficiently remove the modifications on tRNA and has engineered both the 5′ and 3′ ends of the library fragments so that the linker conditions (i.e. 5′ phosphate, 3′ hydroxyl) can be met. Therefore, we specifically built a whitelist in tsRFinder tools with the addition of high confidence tsRNA results identified by PANDORA-Seq as well as other experimental methods. If users use the data obtained by these special methods to predict tsRNA, they can judge whether the predicted tsRNAs are in the whitelist, thereby increasing credibility. While for those researchers who use traditional small RNA-seq library construction method can also learn which important tsRNA molecules may be missed in their data.

In addition, due to the presence of chemical modifications on tRNA, it can cause unexpected stops during the reverse transcription process, or cause mismatches. Therefore, the obtained tsRNA may have false positives. Although studies have investigated that the modification on tRNA has little effect on the identified tsRNAs (33). The main reason is that the modification on the tRNA will cause the reverse transcription to be unable to extend to the linker sequence at the 5′ end, and the corresponding fragment will not be obtained in the subsequent PCR amplification step. However, considering that chemical modification may also cause reverse transcription pauses and generate deletion or mismatch on the sequencing reads, we specifically collected the known modification sites on tRNA and designed a set of penalty strategies accordingly. When there is a mismatch or insertion or deletion (indel), we set different penalty strategies according to whether the site is a known modification site. For example, in normal conditions, a mismatch or indel will subtract 1 from the total score, however, if a mismatch or indel occurs on the chemical modification site, a score of –0.5 is added to the total score of this site. If a perfect match occurs on this site, a score of +1 is added to the total score. We use the score as a parameter for users to choose, and users can adjust the number of potential tsRNAs identified by changing the value of the score.

### Identification of tsRNA targets from CLIP and CLASH/CLEAR data

tsRTarget specializes in predicting tsRNA targets from CLIP-seq datasets. Many studies identified the relationship between miRNAs and mRNAs (11), but they do not predict the target mRNAs of tsRNAs. We designed two pipelines of analysis strategies for different methods of the CLIP-seq library construction (Supplementary Figure S2). In addition to the minimal binding length/strength, we also considered the evolutionary conservation of the tsRNA–mRNA binding sites. We retrieved data from multiple alignments of 99 vertebrate genomes with human from UCSC (12) and adopted the bigWigAverageOverBed software (13) to calculate the conservation scores of the binding sites. tsRTarget will only report the results with the conservation score value greater than 0.3, which increased the confidence of tsRNA target genes.

For AGO CLIP data, we used Cutadapt (Version 2.10) and Trim Galore software (Version 0.4.5) to remove adapter sequences and low-quality reads. Reads of less than 14 nt were discarded. We then collapsed reads with the same sequence and determined the number of each unique read. Reads that aligned to the same strand as the source tRNA transcripts were considered tsRNA fragments. We then obtained the candidate targets that were aligned to the mRNAs and called peaks with the CTK protocol (Version 1.1.3), which calculates the number of overlapping CLIP tags at each genomic position to find local maxima (34). We used RNAhybrid (-c -b 1 -u 2 -v 2 -f 2,7 -n 40 -e -10 -m 70 -s 3utr_human) (35) and BLAST (-word_size 6, mismatch ≤ 2, Version 2.10.1) (36) to search for pairs between candidate tsRNAs and targets.

From the CLASH/CLEAR data, we identified the candidate tsRNA-target chimeras with the basic bioinformatic analysis strategy developed for miRNA-targets in the CLASH technique (21,37). First, we used Bowtie and BLAST software programs to map the candidate chimeras to the genome and remove fake chimeras that mapped to other sites (Supplementary Figure S3). Next, we mapped the reads to the tRNA reference and kept the sequences that partially matched the tRNAs reference (matched read of 14–40 nt and unmatched reads > 8 nt). Then, the candidate tsRNA-target chimeras were split into tsRNAs and target sequences. Duplex structure predictions for the tsRNAs and target regions were made using RNAhybrid and BLAST.

### tsRNA survival analysis and ceRNA network analysis

tsRFun displayed the survival log-rank *P* values of tsRNA molecules in the ‘tsRSurvival’ module, and Kaplan–Meier survival plots were used to visualize the performance of tsRNAs in cancers. We applied two normalization methods in this project. In each cancer data set, we evaluate the abundance of tsRNA by normalizing its count number to the total number of counts that mapped to tRNA:

(2) $$\begin{equation*}RPM\; = \;\frac{{{{10}^6}{\rm{C}}}}{N}\end{equation*}$$

where C represents the count number of a tsRNA, and N represents the total number of all counts that mapped to tRNA. As for pan-cancer analysis, referring to the research of Galka-Marciniak *et al.* (38), we performed a rank-based inverse-Gaussian transformation on the RPM value, and then divided the results by their maximum absolute value, eventually normalized the RPM value of each cancer to the same range [-1,1] with a zero median.

We developed two analysis tools (‘tsRNetwork’ and ‘tsRTarget’) for users to identify interaction networks among tsRNAs, miRNAs, and mRNAs. First, we screened the tsRNA-mRNA and miRNA-mRNA interaction pairs and found a group of tsRNA–miRNA pairs that targeted the same mRNA. Next, a hypergeometric test (18) was used to determine whether a miRNA-tsRNA pair significantly forms a ceRNA pair, based on the number of shared mRNA targets that can interact with both of them. The test calculates the *P* value by using the following formula:

(3) $$\begin{equation*}P\; = \;\sum\nolimits_{i\; = \;k}^{{\rm{min}}\left( {K,n} \right)} {\frac{{{\rm{C}}_K^i{\rm{C}}_{N - K}^{n - i}}}{{{\rm{C}}_N^n}}} \end{equation*}$$

where (i) *N* is the total number of mRNAs used to predict targets, i.e. the number of all human mRNAs; (ii) *K* is the number of mRNAs that interact with the miRNA; (iii) *n* is the number of mRNAs that interact with the tsRNA and (iv) *k* is the number of common mRNAs between these two RNAs. The function p.adjust in the stats package was used to correct the *P* values, with the ‘method’ argument set to ‘FDR’. Finally, all pairs with FDR <0.05 were considered ceRNAs and displayed on the tsRFun page. In this study, we identified ∼10 000 ceRNA pairs from 405 CLIP-seq datasets.

### Database implementation

tsRFun was built with MySQL (Version 5.7.26), PHP (Version 7.1.11), Apache (Version 2.4.39), and JavaScript. Several libraries were used in the process: Bootstrap (Version 4.5.0) controls the layout and style; jQuery (Version 3.5.1) facilitates the dynamic interactions on web pages; dataTable (Version 1.10.22) presents the analysis results as a data frame with paging, filtering, and searching functions; Highchart (Version 8.2.2) visualizes the analysis results in different ways; and GSEA (Version 4.1.0) (39) builds the gene set enrichment analysis. The 15 types of gene sets were downloaded from version 6.2 of the MSigDB database (40) (Supplementary Table S1). The secondary structure of tRNA was displayed with the forna tool of JavaScript (41). tsRFun was designed for multiple browsers, including Google Chrome (17 and later), Firefox (10 and later), Apple Safari (6 and later), and Internet Explorer (9 and later). We have uploaded the open-source code to Github and built a runnable pipeline, to enable our users to perform offline analysis with our tools.

## RESULTS

### Overview of the tsRFun platform

tsRFun aims to provide an online platform for tsRNA identification, target prediction, functional enrichment analysis, as well as pan-cancer expression profiling and survival analysis of tsRNAs across 32 types of cancers. tsRFun consists of six major components: tsRinCancer, tsRSurvival, tsRNetwork, tsRFinder, tsRTarget and tsRFunction. In this section, we provide a complete description of the tsRFun modules.

The tsRFun platform contains three database models (tsRinCancer, tsRSurvival, tsRNetwork) and three web server modules (tsRFinder, tsRTarget, tsRFunction). tsRinCancer integrates the tsRNA expression patterns of 10 572 samples from 32 cancer types. tsRSurvival evaluates the prognostic value of tsRNAs in cancers by the log-rank test and univariate cox-regression. tsRNetwork establishes the ceRNA networks among tsRNAs, miRNAs, and mRNAs by hypergeometric tests. The tsRFinder tool can identify existing tsRNAs in small RNA sequencing data uploaded by users. From CLASH/CLEAR or CLIP sequencing data input by users, tsRTarget can identify possible tsRNA–mRNA interactions and potential tsRNA–miRNA competition relationships, and can further construct a ceRNA network with these RNAs. tsRFunction performs target prediction and gene set enrichment analysis on the tsRNAs that users are interested in.

### Functional description of database module tsRFun

#### tsRinCancer

**Figure 2.**
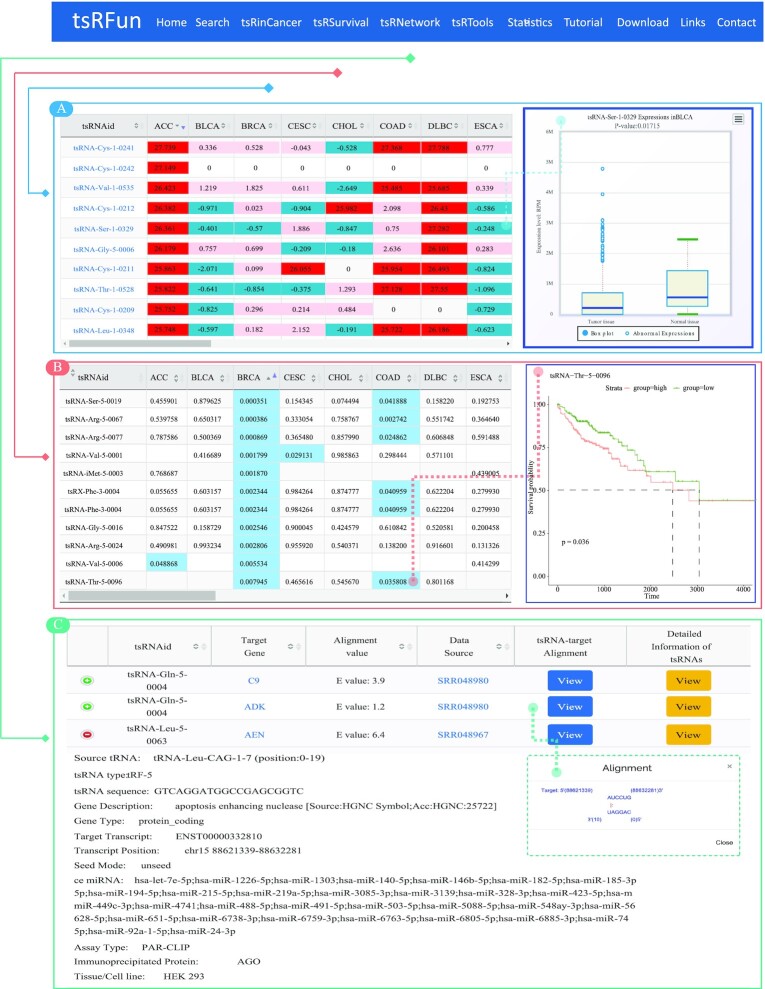
Introduction and usage of tsRFun data presentation. (**A**) tsRinCancer page for tsRNAs with a detailed expression profile across 32 types of cancer. (**B**) tsRSurvival page with the prognostic values of tsRNAs in pan-cancers. (**C**) tsRNetwork page with detailed information of the predicted ceRNA networks among tsRNAs, miRNAs, and mRNAs.

The ‘tsRinCancer’ module investigates the tsRNA expression profiles across pan-cancers types based on 3TB raw small RNA sequencing data. First, we preprocess the raw data and identify a batch of tsRNAs with high confidence by the tsRFinder tool. Then, tsRinCancer provides an integrated view of the tsRNAs across 32 cancer types. tsRinCancer shows the tsRNA expression patterns across these cancers on heatmap plots, and users can browse the differentially expressed tsRNAs between tumour and normal samples with boxplots (Figure 2A).

#### tsRSurvival

The ‘tsRSurvival’ module evaluates the prognostic value of tsRNAs in pan-cancer datasets. In detail, samples are divided into high and low expression groups according to the mean expression level of each tsRNA molecule. Then, the significance of overall survival is calculated by the Kaplan–Meier method and the log-rank test. The tsRNAs with *P* values < 0.05 are highlighted. Users can download the image of the survival analysis results directly from the tsRSurvival page (Figure 2B).

#### tsRNetwork

The ‘tsRNetwork’ builds the interaction networks among tsRNAs, miRNAs, and mRNAs from 405 CLIP data by hypergeometric test (Figure 2C). The ‘tsRNetwork’ module can mine the relationship between tsRNAs and their target genes from CLIP and CLASH/CLEAR data. According to the paired region location pattern, tsRNetwork obtains the canonical and noncanonical binding results, and users can browse the target genes for each tsRNA and the paired structure pattern on the tsRNetwork page. tsRNetwork also displays the details of tsRNAs and the target molecules, as well as the description of the experiment type.

### Functional description of the web server module tsRFun

We developed the tRF2Cancer web server in 2016 (29). The updated tsRFun platform features a series of improvements and enhancements based on the original ‘tsRFinder’ tool and provides two novel analysis tools ‘tsRTarget’ and ‘tsRFunction’ (Table 1).

**Table 1.** Major improvements of tsRFun compared to tRF2Cancer

Data features/functionalities	tRF2Cancer	tsRFun
Clear/clash data	None	Yes
CLIP data	None	Yes
Small RNA seq data format	FASTA	FASTA/FASTQ
tsRNA identification	tRFs (‘tRFfinder’)	tRFs and tiRNAs (‘tsRFinder’)
tsRNA pan-cancer analysis	None	Yes
tsRNA targets prediction	None	Yes (‘tsRTarget’)
tsRNA expression profile	1000 tRFs	∼5000 tsRNAs
tsRNA and miRNA competition relationships	None	Yes
tsRNA Function enrichment	None	15types of gene sets (‘tsRFunction’)

#### tsRFinder

**Figure 3.**
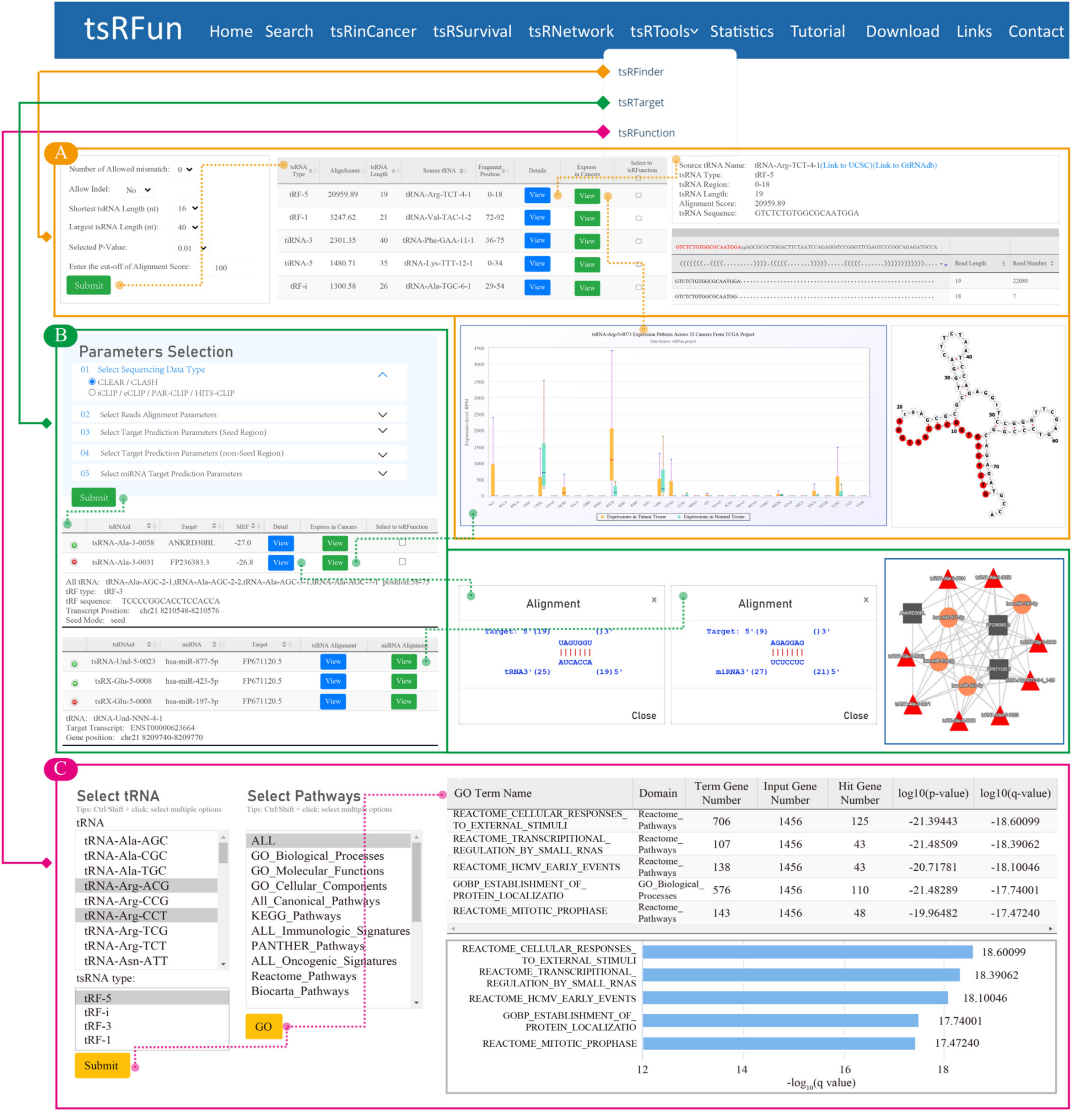
Introduction and usage of tsRFun online tools. (**A**) tsRFinder page for the tsRNAs identification of small RNA seq data with detail type and expression pages. (**B**) tsRTarget page for the tsRNAs targets prediction of CLASH/CLEAR and CLIP seq data with detail tsRNAs information, alignment, and competitive endogenous RNA network. (**C**) tsRFunction page for tsRNAs function prediction.

The ‘tsRFinder’ module allows users to input or upload small-RNA seq data in FASTQ/FASTA format to conduct tsRNA identification analysis with high sensitivity. Parameters such as the number of allowed mismatches, tsRNA length range, and *P* value can be specified by users to narrow the tsRNA prediction results. The tsRNAs analysis results are shown in a table with detailed information including the tsRNA type, tsRNA length, source tRNA information, and fragment position (Figure 3A). Users can sort the data table by column and download the file in Excel or CSV format. More information on the tsRNA, such as the read sequencing distribution, visualization of the read distribution on the source tRNA, structure of the source tRNA, and expression pattern across 32 cancer types in TCGA, can be found in the ‘Detail’ and ‘Express in Cancer’ links. In addition, tsRFun allows users to select multiple tsRNAs for further functional prediction. Moreover, users can either copy the results to the clipboard or download the file in Excel or CSV format.

#### tsRTarget

The ‘tsRTarget’ module allows users to input or upload CLIP, CLASH or CLEAR data to predict potential tsRNA-target interactions based on canonical and noncanonical seed patterns. In addition, tsRTarget constructs a competitive endogenous RNA network with analysis results generated from CLIP data input by users (Figure 3B).

#### tsRFunction

The ‘tsRFunction’ module integrates a list of tsRNA-mRNA interactions from obtained CLIP, CLASH, and CLEAR data, and provides a real-time functional enrichment analysis feature for gene ontology annotation of tsRNAs in 15types of gene sets, including GO, KEGG, Reactome, PANTHER, etc. The default number of highest-ranked gene set enrichment results is 20 (Figure 3C).

### Comparison with other tsRNA databases and web server

There are several databases and online tools for tsRNA investigation, including tRFdb (42), MINTbase 2.0 (43), tRFexplorer (44), tsRBase (45), tRFtarget (46), tRFTar (47) and tRF2Cancer (29). Among these, tRFdb was the first tRF database with a total of 12 877 tRFs from over 100 small RNA libraries, but tRFdb does not store tiRNA molecules and has not been updated since 2015. MINTbase 2.0 and tRFexplorer focuses on tsRNA expression patterns across human cancer types. However, MINTbase 2.0 does not contain tsRNAs derived from tRNA precursors (tRF-1) and tRFexplorer does not include tiRNAs molecules (tiRNA-5 and tiRNA-3) and tRF-i molecules. tRF2Cancer only aimed to identify tRF molecules and has not been updated since 2016.

Many researchers have found a large number of Argonaute-tsRNA complexes in CLIP-seq data (19,20) and revealed that tRF-5 and tRF-3 molecules can bind to Argonaute proteins by seed-based canonical target recognition. Hence tsRBase, tRFtarget, and tRFTar were developed to explore tsRNAs and their targets based on CLIP-seq data. The available web-based and independent tools described above reflected the continuing interest in tsRNAs in the research community. However, these tools do not allow users to upload high-throughput sequencing data in FASTQ or FASTA format to mine the unique relationships between tsRNAs and their targets. Although direct comparison in terms of scope, functionality, ease of use, and other parameters are challenging and partially subjective, we aimed to provide at least an overview of a selection of commonly used broader analysis tools that are available as both databases and web servers. We thus evaluated multiple features of the tsRNA tools and present the results sorted by the publication date (Table 2). The analysis reveals an expected pattern: the recent databases have a broader scope of functionality than the earlier databases. The tsRFun platform provides comprehensive online analysis capability and includes more tsRNA functional tools.

**Table 2.** Comparison of tsRFun with existing tsRNA databases

Databases/ webserver	tRFdb	tRF2Cancer	MINTbase 2.0	tRFexplorer	tsRBase	tRFtarget	tRFTar	tsRFun
**Year of Establishment**	2015	2016	2018	2019	2020	2020	2020	**2021**
**Comprehensive tsRNA Expression Profilings**	No	No	No	No	No	No	No	**Yes**
**tsRNA Targets and Functions Predictions**	No	No	No	No	Yes	Yes	Yes	**Yes**
**tsRNA Prognostic Value Analysis**	No	No	No	No	No	No	No	**Yes**
**tsRNA**,**miRNA, mRNANetwork**	No	No	No	No	No	No	No	**Yes**
**Number of Dataset Sample**	∼500	∼10 000	Over 10 000	Over 10 000	Over 10 000	∼10 000	∼500	**Over 10 000**
**Types of Datasets**	Small-RNA seq	Small-RNA seq	Small-RNA seq	Small-RNA seq	Small RNA-Seq, CLASH, and CLEAR	CLASH	CLASH and CLEAR	**Small RNA-Seq, CLASH, CLEAR and CLIP-Seq**
**Number of tsRNA** **Classes**	3	4	5	5	6	3	5	**6**
**Online Analysis Tools**	No	Yes	No	No	No	No	No	**Yes**

### Evaluating the performance of tsRFinder and tsRTarget with experimentally validated data and other tools

We further compared the performance of the tsRFinder tool with that of other tsRNA identification tools. There are two previously published programs for tsRNA prediction based on high-throughput sequencing data: SPORTS (48), and MINTmap (49). Of note, neither of them is capable of online prediction, so we downloaded their desktop version programs, employed the same running environment to compare the prediction capabilities of tsRFinder, SPORTS, and MINTmap. We downloaded an independent dataset (SRR3235777) by Seashols-Williams *et al.* at GEO (50) and employed the above tools to predict the potential tsRNA molecules. The results show that SPORTS and MINTmap tools have identified more than 6 000 tsRNAs, while tsRFinder only identified 220 (Table 3).

**Table 3.** A list of experimentally validated tsRNAs detected by MINTmap, SPORTS, and tsRFinder

	# total detected tsRNAs	# experimentally confirmed tsRNAs	Precision	Sensitivity
**MINTmap**	6 552	8	8/6 552	8/17
**SPORTS**	6 945	10	10/6 945	10/17
**tsRFinder**	220	10	10/220	10/17

Why tsRFinder get much fewer results than SPORTS and MINTmap? The reason is that while SPORTS and MINTmap directly report all reads aligned to tRNA, tsRFinder performs a binomial test to all the reads mapped to tRNA sequence, to ensure they are bona fide tsRNA instead of tRNA degraded fragments. To prove this point, we collected 17 experiment-verified tRFs from colon cancer datasets generated by Lee *et al.* as a positive control (10), to compare the performance of these three tools. The result shows that SPORTS and MINTmap detected over 6 000 tsRNAs but only 10 and 8 experimental confirmed tsRNAs among them, respectively. By contrast, 10 out of 220 tsRNAs detected by tsRFinder are verified, indicating tsRFinder has higher precision than other tools (Table 3)

Seven of the 17 tsRNA molecules validated by Lee were not identified by tsRFinder, we then inspected the source locations and abundances of these seven tsRNAs and found that although these tsRNAs were able to be detected in the sequencing data, none of the abundances were sufficiently high. For example, tRF-5005, derived from the 5′ end of tRNA-Gly-GCC-1–3, is 20 nt in length (Supplementary Table S2). We could detect tRF-5005 in the sequencing dataset, but only nine reads were able to match exactly to it. In contrast, there is a 30 nt length read also mapped to the 5′ end of tRNA-Gly-GCC-1–3, with 3 122 in abundance (Supplementary Table S2). tsRFinder tool holds that fragments with higher abundance as tsRNA molecules. Since Lee *et al.* used the clone experiment of prostate cancer cell lines (LNCaP and C4-2), while the dataset of Seashols-Williams *et al.* was obtained by large-scale sequencing of prostate cancer cell lines (P69, M12, M2182), it is reasonable that there is the inconsistency of the fragment abundance between the two datasets, which also suggests that tsRNAs have the characteristics of spatio-temporal specificity. To more accurately evaluate the performance of tsRFinder tool, we further generated simulated small RNA-seq datasets by ART (51), which include 100 positive-reads and 29 727 negative-reads (Supplementary Table S4). These simulated datasets were then processed for tsRNA identification by each of the tools, tsRFinder, MINTmap, and SPORTS (Supplementary Table S5-S8). The results showed that a total of 8 387 tsRNA candidates were identified by MINTmap, including only 61 true tsRNAs, with a low precision rate of 0.73%. Similarly, SPORTS predicted a total of 12 196 tsRNA candidates, including only 88 true tsRNAs, with a low precision rate of 0.72%. While tsRFinder predicted only 99 tsRNA candidates, including 81 true tsRNAs, with a high precision rate of 81.82% (Supplementary Table S5). The above results illustrated that tsRFinder adopted more stringent screening conditions, which greatly increased the precision rate of tsRNA predictions.

In addition, we also compared the predicted targeted genes of tsRTarget with tRFTar tool (16) (Supplementary Table S3). The results show that tsRTarget and tRFTar tools have identified > 5 000 tsRNAs-target interactions, while the overlap is 86.69% (Supplementary Figure S4). Due to the limited research that has verified the interaction between tsRNAs and mRNAs (17), it is difficult to evaluate the sensitivity/specificity. We list the CLIP-Seq experiment ID on which the prediction result is based, and show their detailed sequence alignment between tsRNAs and mRNAs, so that users can verify each result. We will continue to pay attention to this field, looking for more verified target relationships, to evaluate the sensitivity/specialty of tsRNA target prediction tools.

## DISCUSSION

tsRNAs are a class of newly discovered noncoding RNA molecules that play important roles in physiological and pathological processes. Notably, the abundance of tsRNA molecules in cells is comparable to that of miRNAs. However, current tsRNA databases have a series of limitations, including a lack of recent updating, incomplete data on tsRNA types (tRFs or tiRNAs), and missing information on the interactions between tsRNAs and their targets. Most importantly, online tools that can analyse the function of tsRNAs and their targets in user-provided high-throughput sequencing data have not been developed thus far, making it difficult to meet the growing demand of tsRNA research.

tsRFun is currently a unique platform containing tsRNA databases and real-time online web server tools. We improved tsRFun from four perspectives (systematicity, accuracy, sensitivity, and efficiency), and developed an integrated systematic online analysis platform for tsRNA identification, target prediction, pan-cancer expression profiling, and functional enrichment. tsRFun enables specific tsRNA analysis from high-throughput sequencing data based on user-selected threshold parameters. Previous research approaches have attempted to identify the unified functions and mechanisms of action from the categories of tsRNAs. However, studies have revealed that the members of the same class of tsRNAs may perform quite different functions. In this project, we constructed a regulatory network based on the relationships between tsRNAs and their targets and then evaluated the similarity of each gene function in the same network module to predict the function of tsRNAs. tsRFun will expand our understanding of tsRNA functions and reveal the roles of tsRNAs in the onset and progression of cancers. This study performed a pan-cancer analysis of tsRNAs in 32 cancers and identified a batch of tsRNA molecules that are closely associated with cancer development and progression. tsRFun integrates sequencing data from three aspects—transcriptomics, RNA modification-omics, and RNA protein interactomics—to expand large-scale studies of tsRNAs and their associated functions from a multidimensional, high-throughput perspective.

tsRFinder showed stronger precision due to the binomial test employed by tsRFinder to determine whether the fragments obtained from sequencing data were real tsRNAs, not degraded fragments of tRNAs, while other tools consider all sequenced fragments capable of mapping onto tRNA transcripts as potential tsRNA molecules. Instead of consuming much time and effort on one degraded fragment, we believe our tool can better assist biologists in selecting the truly meaningful tsRNAs. We have demonstrated that tsRFinder has good sensitivity and better precision than other tsRNA prediction tools, but we cannot give specificity information of tsRFinder or any tsRNA prediction tools at present. Since there is currently no accurate experimental validation negative dataset on tsRNA, therefore cannot calculate true negatives and false negatives in the prediction results of each tool. Currently, most of the tsRNAs in the available databases are the results of software predictions, and there must be a large number of false positives (tRNA degraded fragments). Therefore, our purpose in establishing tsRFun was to provide a batch of high confidence tsRNAs and their expression patterns in tumours. At the same time, we also caution researchers within the field that one cannot consider a stretch of tsRNAs solely on the basis of sequencing results being able to match to a tRNA transcript. Since we cannot prove the stronger specificity of tsRNAs at present, we modified the description in the manuscript as tsRFinder has good sensitivity and better precision than other tsRNA prediction tools, and added a discussion about specificity. In the future, as more experimentally validated tsRNAs are discovered, we will also continuously refine the dataset of high confidence tsRNAs and thus evaluate the specificity of tsRFun and other tsRNAs identification tools.

Currently, there are few online analysis tools for tsRNA molecules. With the increase in small RNA sequencing data (especially large-scale tumour related datasets), it is necessary to develop a series of convenient and effective tools to explore the expression distribution patterns and potential biological functions of tsRNA molecules in diseases and cancers. Therefore, we developed the tsRFun online platform to meet the research needs for analysis of tsRNA molecules, including systematic identification of various classes of tsRNAs, investigation of the expression patterns of tsRNAs in multiple cancer types, and finding out important tsRNAs associated with cancers. In addition, this study identified the interaction relationships between tsRNAs and protein-coding genes based on AGO CLIP-Seq data and established a coexpression network to predict the function of tsRNA molecules. In summary, this study integrates databases, computational methods, and analytical techniques to develop an effective analytical tool for tsRNA research, providing strong evidence for comprehensively revealing the roles of tsRNA in physiological and pathological processes.

## CONCLUSION

As a novel type of regulatory small RNA, tsRNA has expanded the research field of noncoding RNAs. Although researchers' current understanding of tsRNA is not comprehensive, tsRNAs play indispensable regulatory roles at many biological levels. The tsRFun platform developed in this study is a systematic and comprehensive platform of tsRNAs that facilitates the investigation of known and novel tsRNAs and predicts their functions, promising to advance subsequent studies of tsRNAs.

## DATA AVAILABILITY

tsRFun is freely available at http://rna.sysu.edu.cn/tsRFun/ or http://biomed.nscc-gz.cn/DB/tsRFun/. We have deposited the related codes on GitHub (https://github.com/zhlingl/tsRFun) to facilitate more users to use our tools.

## Supplementary Material

gkab1023_Supplemental_File
