# Supplementary material for: tsRFun: a comprehensive platform for decoding human tsRNA expression, functions and prognostic value by high-throughput small RNA-Seq and CLIP-Seq data
Source: Nucleic Acids Res. 2021 Nov 10;50(D1):D421–31. doi: 10.1093/nar/gkab1023 (PMC8728237; doi:10.1093/nar/gkab1023)
Supplement: gkab1023_Supplemental_File [file gkab1023_supplemental_file.pdf]

Supporting Information

tsRFun: a comprehensive platform for decoding human tsRNA expression, functions and prognostic value by high-throughput small RNA-Seq and CLIP-Seq data

Authors:

Jun-Hao Wang[1,3], Wen-Xin Chen[1], Shi-Qiang Mei[1], Yue-Dong Yang[2], Jian-Hua Yang[1,*], Liang-Hu Qu[1,*], Ling-Ling Zheng[1,*]

Affiliation:

[1]Key Laboratory of Gene Engineering of the Ministry of Education, State Key Laboratory for Biocontrol, Sun Yat-sen University, Guangzhou 510275, P. R. China

[2]National Supercomputer Center in Guangzhou, Sun Yat-sen University, Guangzhou, China

[3]School of Medicine, Sun Yat-sen University, Shenzhen, Guangdong, China.

*Corresponding author:

Mailing address: Biotechnology Research Center, Sun Yat-sen University, Guangzhou 510275, P. R. China

Phone: 86-20-84112399

Fax: 86-20-84036551

Jian-Hua Yang, Email: yangjh7@mail.sysu.edu.cn

Liang-Hu Qu, Email: lssqlh@mail.sysu.edu.cn

Ling-Ling Zheng, Email: zhengll33@mail.sysu.edu.cn

Supplementary Figures

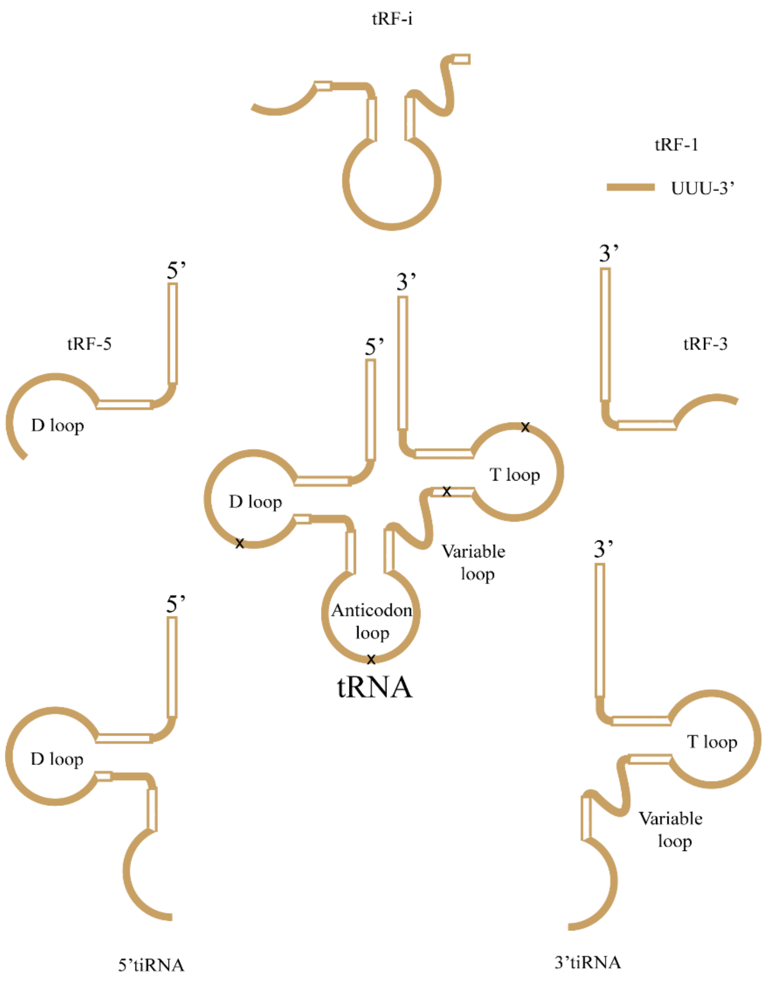

Figure S1. Biogenesis and classification of tRFs and tiRNAs. tRF-1 is generated from precursor tRNA and cleaved by RNase Z or ELAC2 at 3' trailer. tRF- 3 and tRF-5 are generated from the 3' and 5' ends of the mature tRNAs, respectively. tRF-i is mainly from the internal region of mature tRNA. tiRNAs are generated by specific cleavage by angiogenin in the anticodon loops of mature tRNAs.

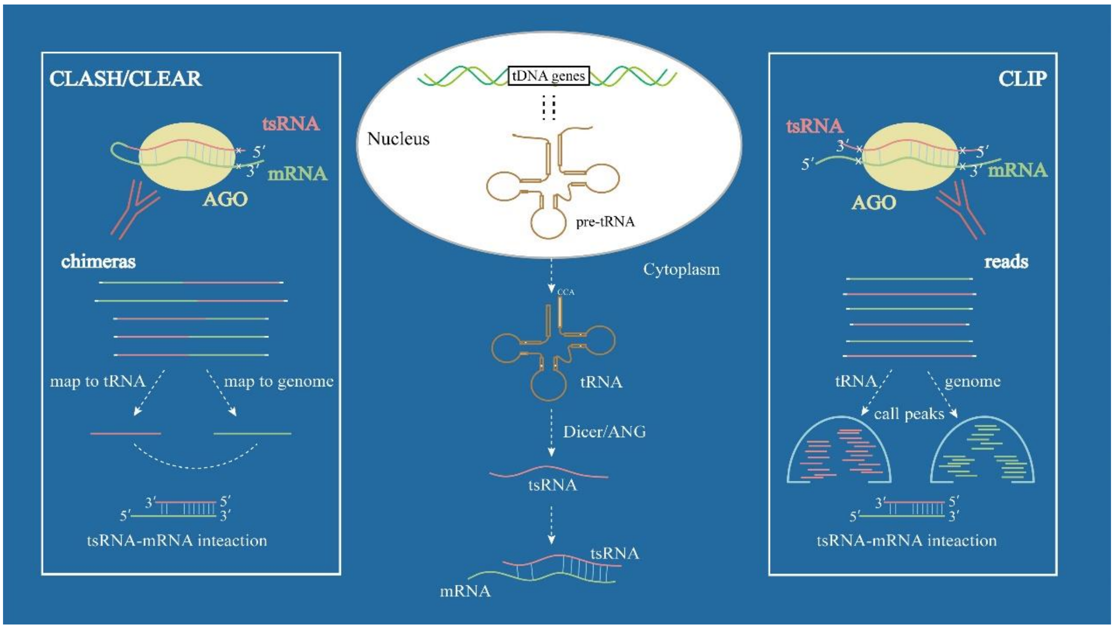

Figure S2. Schematic diagram of tsRNA target identification. For CLASH/CLEAR data (left), we match the chimeric reads to tRNA and genome reference respectively, and then detect their complementary pairing relationship. For CLIP data (right), we match the reads to tRNA and genome reference, then call peaks to find enriched clusters, and check the complementary pairing relationship between these clusters.

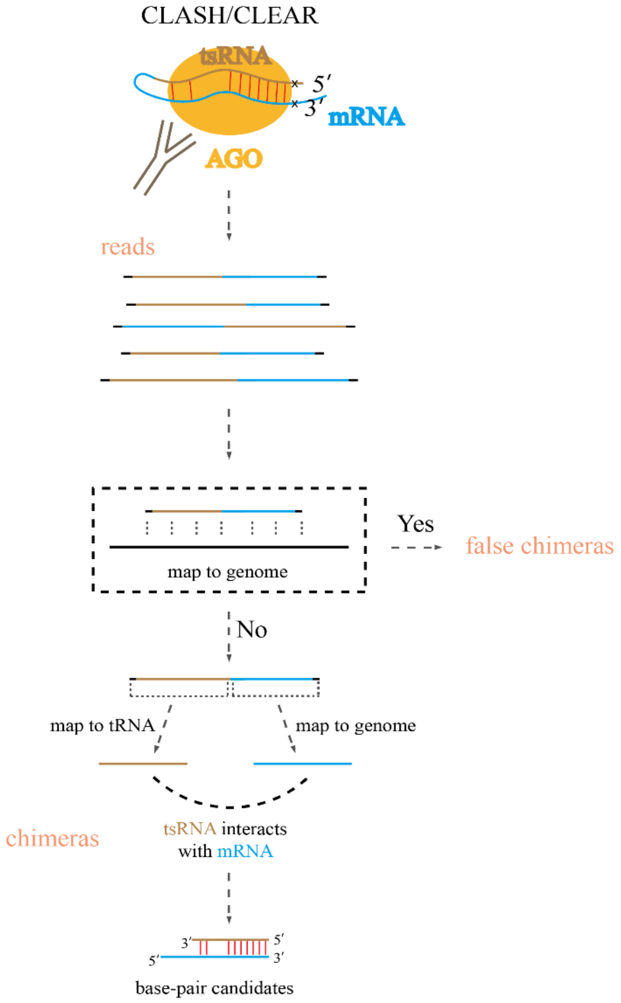

Figure S3. The analysis strategy to identifying tsRNA-target chimeras.

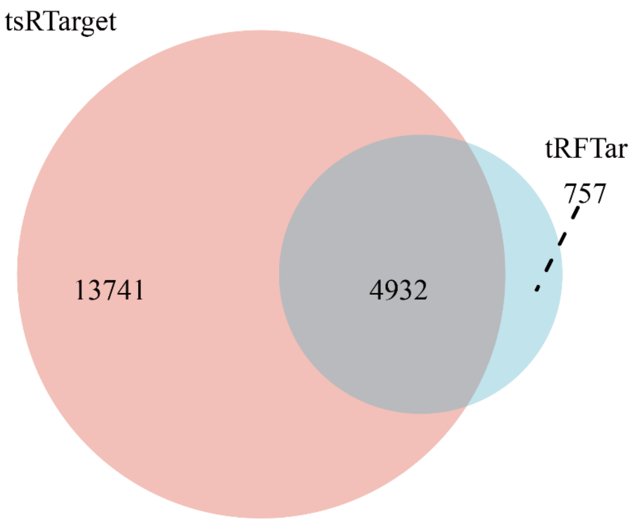

Figure S4. Venn diagram of tsRNA-mRNA chimera results identified by tsRTarget and tRFTar from CLASH/CLEAR and CLIP-Seq data.

Supplementary Tables

Table S1. A list of gene sets used in gene enrichment analysis.

ID	Name
1	All Canonical Pathways
2	All Immunologic Signatures
3	All Oncogenic Signatures
4	BioCarta Pathways
5	Cancer Gene Neighborhoods
6	Chemical and Genetic Perturbations
7	Disease Ontology
8	GO Biological Processes
9	GO Cellular Components
10	GO Molecular Functions
11	Hallmark GeneSets
12	KEGG Pathways
13	PANTHER Pathways
14	Reactome Pathways
15	Transcription Factor Targets

Table S2. Alignment of sequencing reads to tRNA-Gly-GCC-1-3 transcript. The first two lines of the table indicate the sequence and the RNA secondary structure of tRNA. Each next row represents a sequencing read and its corresponding position on the tRNA. The asterisk indicates the read identified as tsRNA by the corresponding tool or article.

tRNA-Gly-GCC-1-3 sequence	Read Length	Abundance	Lee et al.	tsRFinder	Other Tools
GCATGGGTGGTTCAGTGGTAGAATTCTCGCCTGCCACGCGGGAGGCCCGGGTTCGATTCCCGGCCCATGCACCA					
(((((((..((((.......)))).(((((.......))))).....(((((.......))))))))))))....					
GCATGGGTGGTTCAGTGGTAGAATTCTCGCCTGCCACGCGGGAGG.............................	45	3			*
GCATGGGTGGTTCAGTGGTAGAATTCTCGCCTGCCACGCGGGAG..............................	44	2			*
GCATGGGTGGTTCAGTGGTAGAATTCTCGCCTGCCACGCGGGA...............................	43	2			*
GCATGGGTGGTTCAGTGGTAGAATTCTCGCCTGCCACGCGGG................................	42	7			*
GCATGGGTGGTTCAGTGGTAGAATTCTCGCCTGCCACGCGG.................................	41	1			*
GCATGGGTGGTTCAGTGGTAGAATTCTCGCCTGCCACGCG..................................	40	19			*
GCATGGGTGGTTCAGTGGTAGAATTCTCGCCTGCCACG....................................	38	9			*
GCATGGGTGGTTCAGTGGTAGAATTCTCGCCTGCCAC.....................................	37	24			*
GCATGGGTGGTTCAGTGGTAGAATTCTCGCCTGCCA......................................	36	106			*
GCATGGGTGGTTCAGTGGTAGAATTCTCGCCTGCC.......................................	35	544			*
GCATGGGTGGTTCAGTGGTAGAATTCTCGCCTGC..	34	37			*
GCATGGGTGGTTCAGTGGTAGAATTCTCGCCTG...	33	370			*
GCATGGGTGGTTCAGTGGTAGAATTCTCGC..	30	3122		*	*

GCATGGGTGGTTCAGTGGTAGAATTCTCG...	29	148		*
GCATGGGTGGTTCAGTGGTAGAATTCTC..	28	85		*
GCATGGGTGGTTCAGTGGTAGAATTCT...	27	30		*
GCATGGGTGGTTCAGTGGTAGAATTC..	26	8		*
GCATGGGTGGTTCAGTGGTAGAATT...	25	20		*
GCATGGGTGGTTCAGTGGTAGAAT..	24	8		*
GCATGGGTGGTTCAGTGGTAGAA...	23	4		*
GCATGGGTGGTTCAGTGGTAGA..	22	15		*
GCATGGGTGGTTCAGTGGTAG...	21	5		*
GCATGGGTGGTTCAGTGGTA..	20	9	*	*
GCATGGGTGGTTCAGTGGT...	19	9		*
GCATGGGTGGTTCAGTGG..	18	15		*
GCATGGGTGGTTCAGTG...	17	2		*
GCATGGGTGGTTCAGT..	16	7		*
GCATGGGTGGTTCAG...	15	6		*

Table S3. A list of tsRNAs-target interactions detected by tsRTarget and tRFTar tool.

	tsRTarget	tRFTar	overlap
**Targeted gene number**	18,673	5,689	4,932

Table S4. The positive and negative sets in the simulated dataset.

	Read number	Read abundance
**Positive set**	100	82,027
**Negative set**	29,727	75,880
**Total**	29,827	157,907

Table S5. The performance among MINTmap, SPORTS, and tsRFinder tools.

	Precision (%)	Sensitivity (%)	Specificity (%)	False positive rate (%)	False negative rate (%)	Accuracy (%)
**tsRFinder**	81.82	81	99.94	0.06	0.19	99.86
**MINTmap**	0.73	61	71.99	28.01	0.39	71.95
**SPORTS**	0.72	88	59.27	40.73	0.12	59.37

Table S6. The prediction result of tsRFinder tool.

	Actual_true	Actual_false	Total
**Predicte_true**	81	18	99
**Predicte_false**	19	29,709	29,728
**Total**	100	29,727	29,827

Table S7. The prediction result of MINTmap tool.

	**Actual_true**	**Actual_false**	**Total**
**Predicte_true**	61	8,326	8,387
**Predicte_false**	39	21,401	21,440
**Total**	100	29,727	29,827

Table S8. The prediction result of SPORTS tool.

	**Actual_true**	**Actual_false**	**Total**
**Predicte_true**	88	12,108	12,196
**Predicte_false**	12	17,619	17,631
**Total**	100	29,727	29,827
